# Supplementary material for: A pair of atypical NLR-encoding genes confers Asian soybean rust resistance in soybean
Source: Nat Commun. 2024 Apr 17;15:3310. doi: 10.1038/s41467-024-47611-y (PMC11023949; doi:10.1038/s41467-024-47611-y)
Supplement: Supplementary file 1 — Supplementary Information [file 41467_2024_47611_MOESM1_ESM.pdf]

An pair of an atypical NLR encoding genes confer Asian soybean rust resistance in soybean

Hao *et al.*

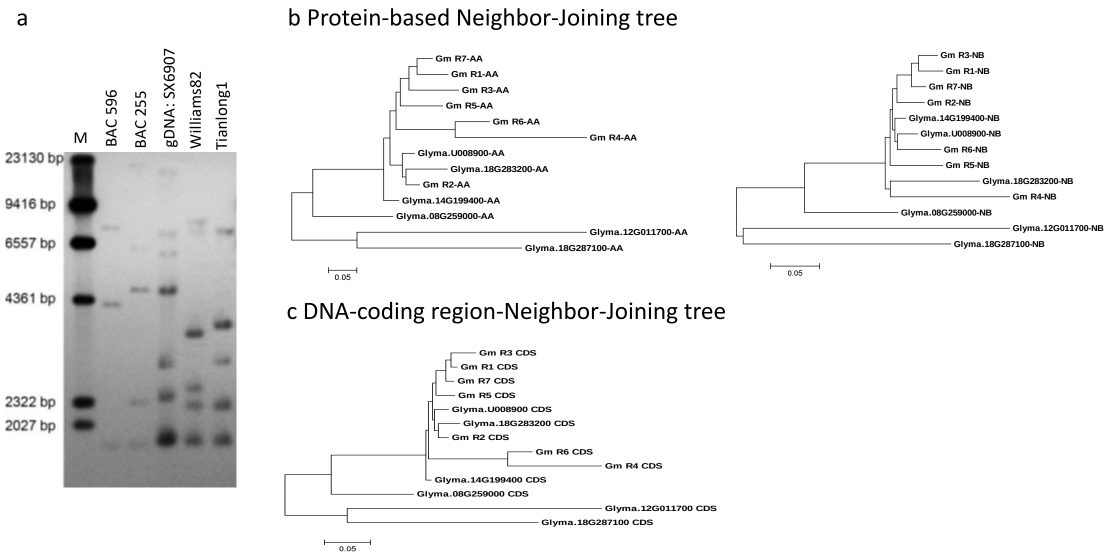

Supplementary Fig. 1. The inserted fragment in the *Rpp6907* locus. a Southern blot of HindIII-digested DNA of BAC 255 and BAC 596 on the identified NLR genes and, as a control, digested genomic DNA from the SX6907, Williams82 and Tianlong1. The DIG labeled probe was designed on the region coding for the so called "P-loop". **b** Neighbor-Joining tree of complete *Rpp6907* locus NLR like proteins, some homologous proteins annotated in the reference genome and CcRpp1 homologous like proteins in soybean. **c** DNA coding regions based Neighbor-Joining tree. Sequences were aligned with Muscle as implemented in Mega 6.0. Mega was also used to generate phylogenetic trees using the pair-wise deletion method. Bootstrap confidence values based on 1,000 iterations are indicated in the nodes.

>Rpp6907-7

MADSVVAFLLDNLSRLLEDEHKLLSGVEDKVNSLCNELKFIHIFLKNSEGKRSHDTVKEVVSQIRDVAH
KAEDVVDTYVTNITRHKQRSKLSKLFHLKEQVMVLHQVNSDIEKIRSQIDDIYKNRDRYGIGEGEFRSE
EAAAEAESLLKRRREVEEEDVVGLVHDSSHVIQELMESESRLKVVSIIGMGGLGKTTLARKIHNNNQVQ
LRFPCLAWVSVSNDYRPKECLLSLLKCSMSSTSEFEKLSEEELKKKVAEWLKEKRYLVVLDDIWETQVW
DEVKGAFPDDQTGSRILITSRNKDVAHYAGTASPYDLPILNEDESWELFTKKIFRGEECPSDLEPLGRS
IVKTCGGLPLAIVVLAGLVAKKEKSQREWSRIKDVSWHLTEDKTGVMDILKLSYNNLPGRLKPCFLYFG
IYPEDYEISPRQLIQYWIAEGFIQPQKTGIADTTELEDVADFYLDELVDRSLVQVAERRSDGGVKTCRI
HDLLRDLCLSESKSDKFLEVCTNSNIDTVSNTNPRRMSIHLKGDSGVSANTFNKSCTRSMFIFGSDDRM
DLVPVLKNFELARVLDCDSFIGFSSYPVPRDLKRMIHLRYLRICVKHLPDCVCSLWNLETLDVTYETTV
SSKIWTLKRLRHLYLSGGGKLPVVLPKANRMENLQTLLLDGSYGIDTYPQQVISLLKIDIFPRLRKLAL
RYPD...GPDMLSSLHHLSNLRSLKVIRGFELPSDTNAFPSNLTKIT..LAAVRDLH.FMKTLGQLTNL
QILKLRFGKVGFHLDIGRGEFPQLQVLHMTQINVRQWRLEKDAMPRLRHLVINDCEELSELPEELWSMT
ALRVVHVSRPSEELANSLKNVEPRNGCKLKISNEL

Supplementary Fig. 2. Natural variation in *Rpp6907-7*. Distribution of amino acids showing evidence of positive selection in Rpp6907-7. Dark grey highlight: coiled-coil domain, light gray highlight: nucleotide binding domain, light blue highlight: leucine rich repeat, and red letters with yellow highlight: amino acids showing significant evidence of positive selection.

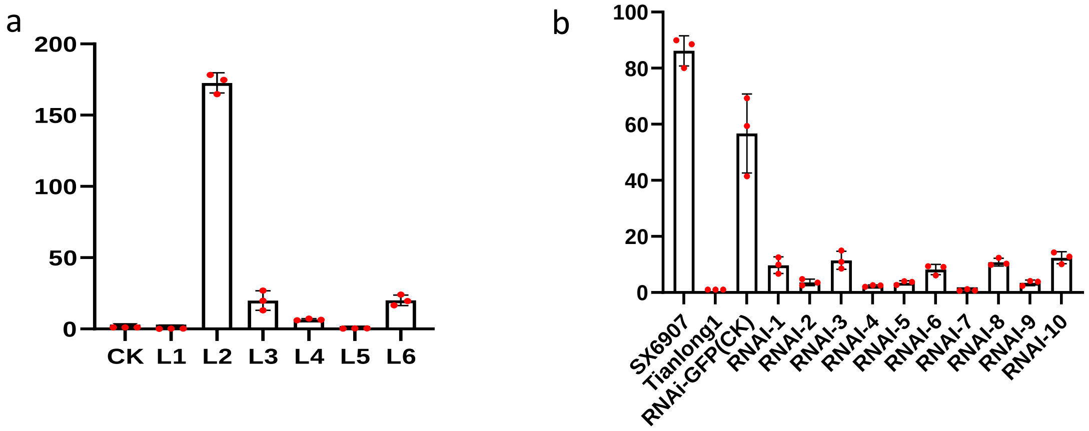

Supplementary Fig. 3. Relative expression levels of *R7* in transgenic lines determined by qRT-PCR analysis. a Relative expression levels of *R7* in six putative T_0 over-expression transgenic plants. CK: Tianlong1. L1-L6: *R7* transgenic lines. The values are means ± SD (*n* = 3 biological. The Actin gene as an internal reference. **b** Relative expression levels of *R7* in 10 putative T_0 RNAi transgenic plants. The values are means ± SD (*n* = 3 replicates). The Actin gene as an internal reference. Source data are provided as a Source Data file.

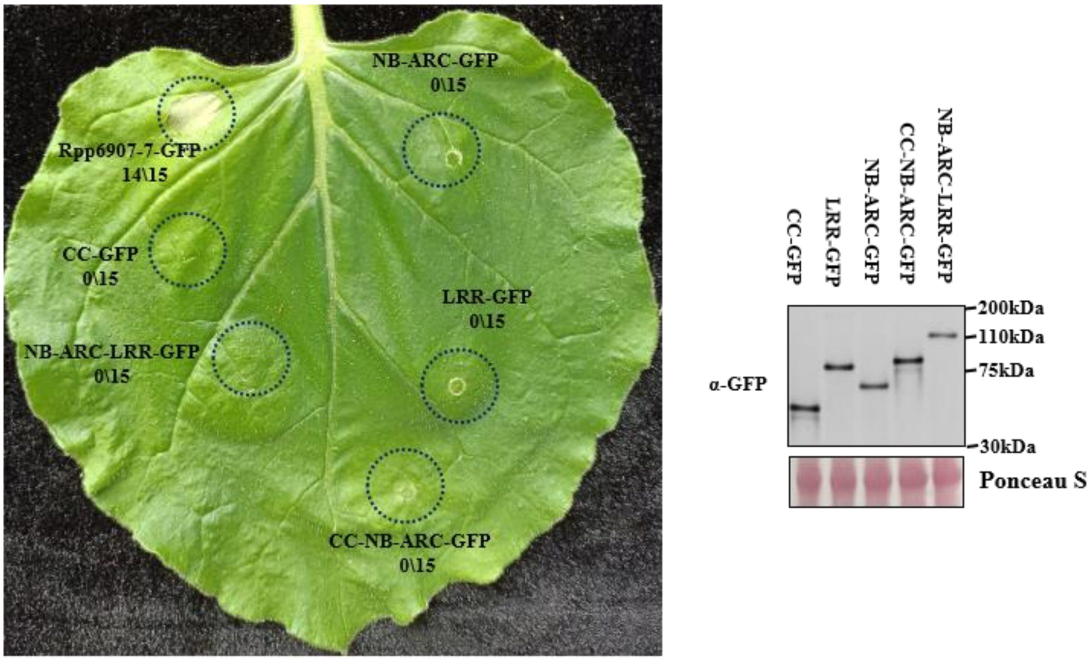

Supplementary Fig. 4. HR assays of different domains of Rpp6907-7 in *N. benthamiana*. Immune signaling is induced by activated full-length Rpp6907-7 only. HR was not induced by Rpp6907-7 fragments or full-length Rpp6907-7 transiently expressed in *N. benthamiana*. The different domains of Rpp6907-7 fusion proteins were transiently expressed in *N. benthamiana*. The strain carrying all constructs was infiltrated at an OD600 of 0.3. HR induced by the different constructs was visualized 4 days after infiltration.The numbers in paren theses indicate the numbers of leaves displaying cell death out of the total number of leaves infiltrated. The representative images from a single replicate of three independent experiments are shown. Western blots probed with anti-GFP antibodies show the typical expression of all tested proteins. Expected protein size was 53.1 kDa for CC:GFP, 79.8 kDa for LRR:GFP, 63.3 kDa for NB-ARC:GFP, 82.4 kDa for CC-NB-ARC:GFP, 111.0 kDa for NB-ARC-LRR:GFP. Modified ponceau staining is shown to indicate loading. Source data are provided as a Source data file.

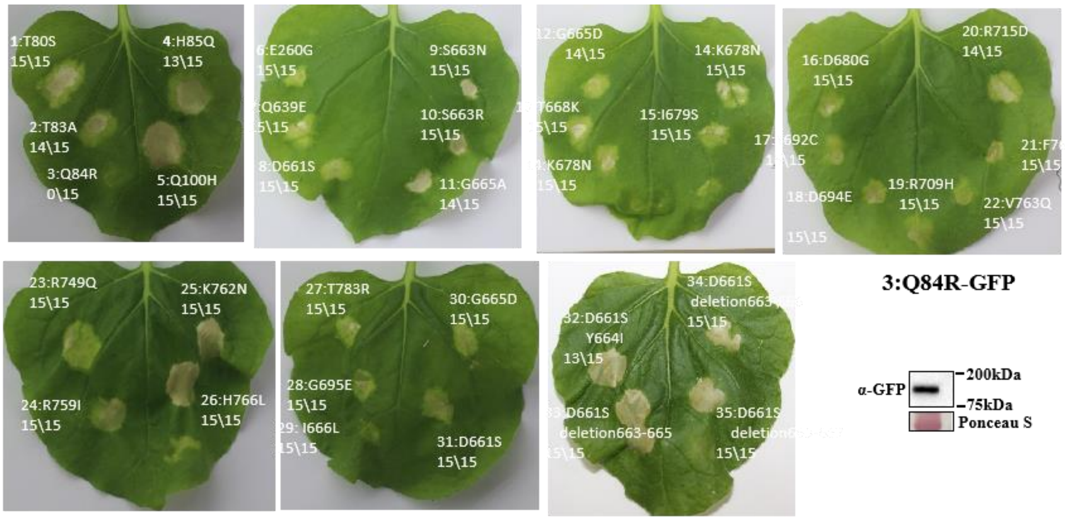

Supplementary Fig. 5. Screening of special amino acids of Rpp6907-7 that are critical for the induction of cell death. The different mutants of Rpp6907-7 GFP fusion proteins were transiently expressed in *N. benthamiana*. HR was not induced by Rpp6907-7Q84R. The strain carrying all constructs was infiltrated at an OD600 of 0.3. HR induced by the different constructs was visualized 5 days after infiltration. The numbers in paren theses indicate the numbers of leaves displaying cell death out of the total number of leaves infiltrated. The representative images from a single replicate of three independent experiments are shown. The utilization of fusion GFP is employed for the purpose of detecting the typical expression of the target protein. The letter represents the amino acid abbreviation, and the number between the two letters represents the amino acid position.Western blots probed with anti-GFP antibodies show the protein accumulation of Rpp6907-7Q84R. Expected protein size was 130 kDa for Rpp6907-7Q84R:GFP. Modified ponceau staining is shown to indicate loading. Source data are provided as a Source data file.

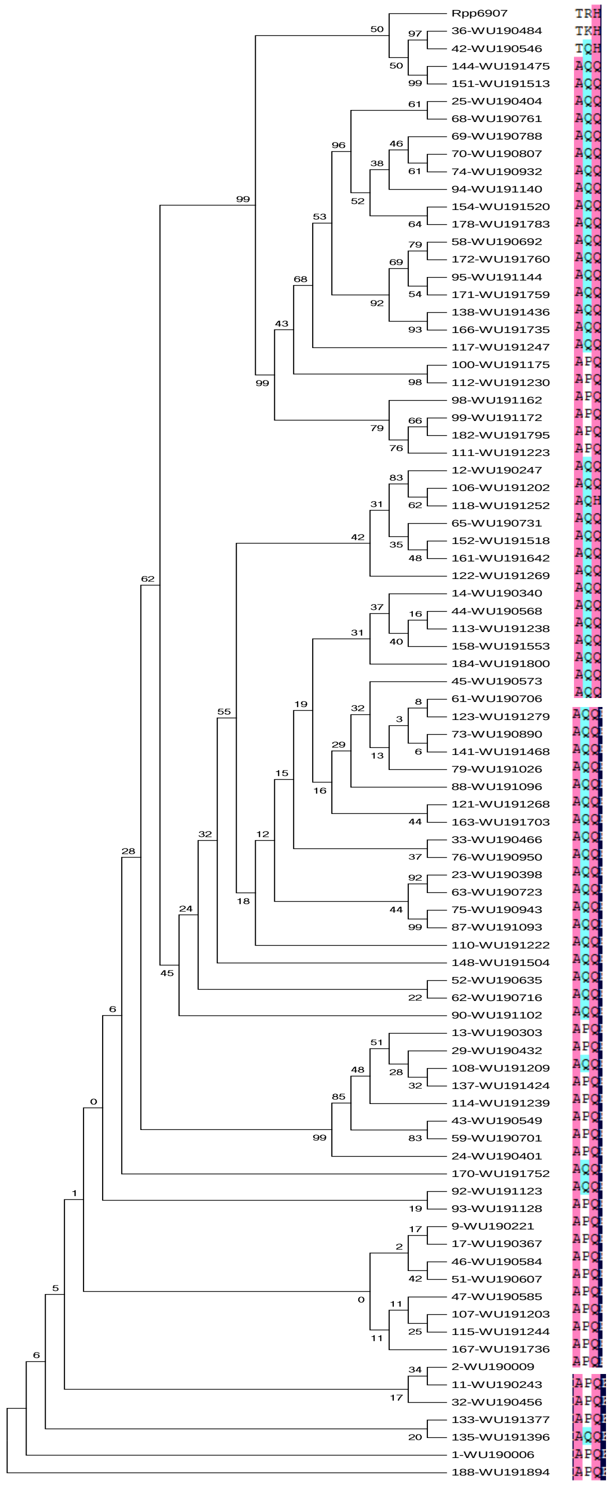

Supplementary Fig. 6. Phylogenetic analysis of *Rpp6907-7*-sequences similar genes in different soybean resources. Neighbor-joining tree of the Rpp6907-7-sequences similar genes identified in a collection of 83 Chinese soybean accessions. The genes were amplified with the primers Rpp6907-7-F and Rpp6907-7-R (Supplementary Data 6). The tree was generated using Mega version 6.0. Bootstrap confidence values based on 1,000 iterations are indicated in the nodes. The letters to the right of the tree indicate the two predicted amino acids affected by site-specific mutation 84, which resulted in no HR in *N. benthamiana.*

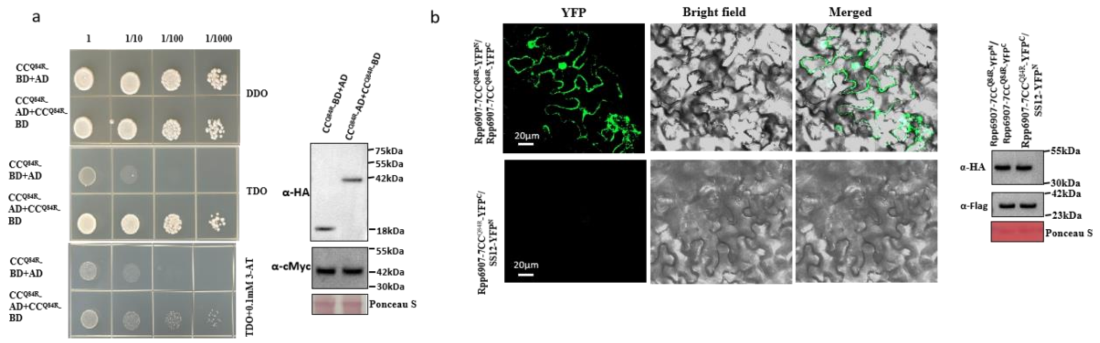

Supplementary Fig. 7. Protein interaction analysis of Rpp6907-7 CC$^{\mathbf{84\ (Q\ to\ R)}}$ **mutation domains. a** Yeast-two hybrid assay reveals interaction of Rpp6907-7 CC84 (Q to R) mutation domain. Dilution series of yeast cells expressing GAL4-AD and GAL4-BD fusions of Rpp6907-7CC^{Q84R} domain on non-selective synthetic media lacking Trp and Leu (DDO) and selective media additionally lacking His (TDO). Photographs were taken after four days of incubation and are representatives of at least three independent experiments with yeast obtained from three independent yeast transformations. Western blots probed with anti-HA and anti-cMyc antibodies show protein accumulation of all tested proteins. Expected protein size was 18.6 kDa for AD, 40 kDa for CC$^{84\ (Q\ to\ R)}$-AD, 43 kDa for CC$^{84\ (Q\ to\ R)}$-BD. Modified ponceau staining is shown to indicate loading. **b** The self-association of CC domains was not lost as a result of the 84 (Q to R) mutation, as determined by BiFC analysis. Unrelated protein SS12 (Glyma.14G199400 1-182 CC domain) as negative controls to test the specificity of the interactions detected. Western blots probed with anti-HA and anti-Flag antibodies show protein accumulation of all tested proteins in BiFC experiments. Expected protein size was 39 kDa for Rpp6907-7CC^{Q84R}-YFP^{N}, 39 kDa for SS12-YFP^{N}, 33 kDa for Rpp6907-7CC^{Q84R}-YFP^{C}. Modified ponceau staining is shown to indicate loading. All images are representative of results obtained from three independent experiments with similar results. Source data are provided as a Source data file.

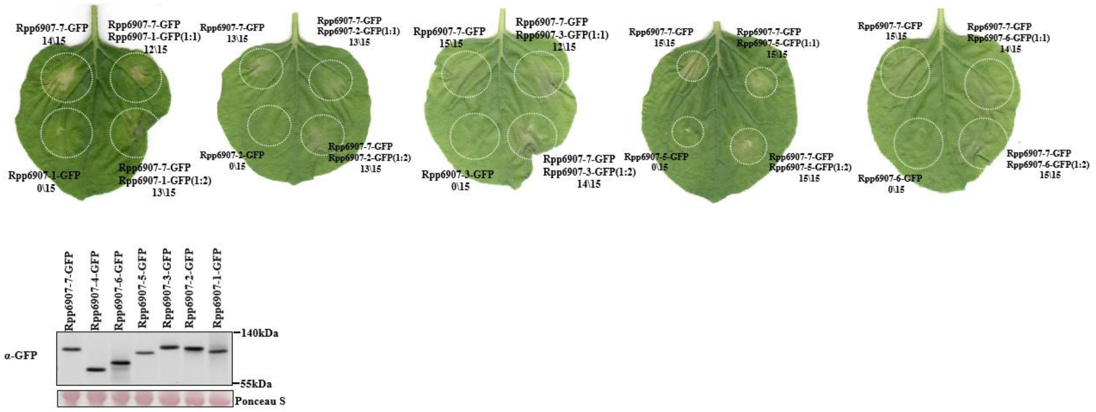

Supplementary Fig. 8. Co-expression of *Rpp6907-7* and other genes in the *Rpp6907* locus (R1 to R6) in *N. benthamiana*. Rpp6907-1, Rpp6907-2, Rpp6907-3, Rpp6907-5, Rpp6907-6 and Rpp6907-7 failed to inhibit the HR induced by Rpp6907-7 in *N. benthamiana* leaves with a ratio of 1:2 and 1:1, respectively. HR induced by the different constructs was visualized 4 days after infiltration.The numbers in paren theses indicate the numbers of leaves displaying cell death out of the total number of leaves infiltrated. The representative images from a single replicate of three independent experiments are shown. The utilization of fusion GFP was employed for the purpose of detecting the typical expression of the target protein. Western blots probed with anti-GFP antibodies show the protein accumulation. Expected protein size was 130 kDa for Rpp6907-7:GFP, 85 kDa for Rpp6907-4:GFP, 94 kDa for Rpp6907-6:GFP, 120 kDa for Rpp6907-5:GFP, 145 kDa for Rpp6907-3:GFP, 138 kDa for Rpp6907-2:GFP, 128 kDa for Rpp6907-1:GFP. Modified ponceau staining is shown to indicate loading. Source data are provided as a Source data file.

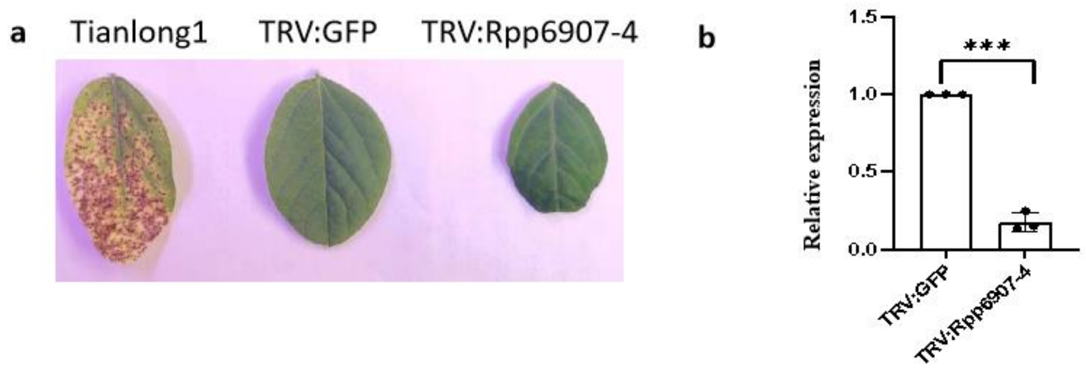

Supplementary Fig. 9. Rpp6907-4 is not required for Rpp6907-7-mediated resistance in SX6907. a Soybean plants were subjected to VIGS by inoculation with TRV constructs (TRV:GFP or TRV:Rpp6907-4). Four weeks after inoculation, GFP and Rpp6907-4 were transiently expressed in the gene-silenced leaves and then SS4 inoculation and resistance identification were carried out. Leaves were photographed 14 d later.The experiment was performed five times with five plants for each TRV construct. Representative photographs are shown. **b** *Rpp6907-4* expression levels after VIGS treatment determined by RT-qPCR analysis. Actin gene was used as the endogenous control. Means and standard errors from three biological replicates are shown. *P* values were calculated with the two-tailed Student's *t*-test. Asterisks indicate significant differences ($P \leqq 0.001$). Source data are provided as a Source data file.

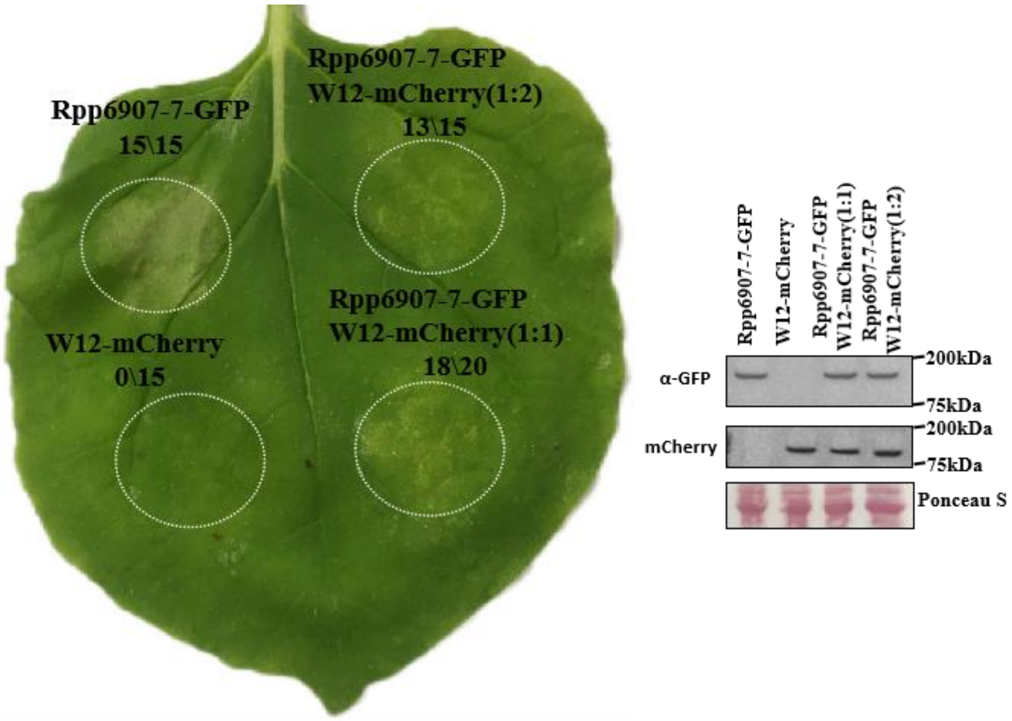

Supplementary Fig. 10. Rpp6907-4-like gene (*W12*) suppresses HR induced by Rpp6907-7. Rpp6907-4-like gene (*W12*) form Tianlong1 suppresses HR induced by Rpp6907-7 in *N. benthamiana* leaves with a ratio of 1:2 and 1:1 between Rpp6907-7 and W12 carrying *Agrobacterium* in the infiltration inoculum, respectively. HR induced by the different constructs was visualized 4 days after infiltration. The numbers in paren theses indicate the numbers of leaves displaying cell death out of the total number of leaves infiltrated. The utilization of fusion GFP and mCherry were employed for the purpose of detecting the typical expression of the target protein. Expected protein size was 130 kDa for Rpp6907-7:GFP, 131 kDa for W12:mCherry. Modified ponceau staining is shown to indicate loading. All images are representative of results obtained from three independent experiments with similar results. Source data are provided as a Source data file.
