# Supplementary material for: A pair of atypical NLR-encoding genes confers Asian soybean rust resistance in soybean
Source: Nat Commun. 2024 Apr 17;15:3310. doi: 10.1038/s41467-024-47611-y (PMC11023949; doi:10.1038/s41467-024-47611-y)
Supplement: Supplementary file 3 — Description of Additional Supplementary Files [file 41467_2024_47611_MOESM3_ESM.pdf]

Description of Additional Supplementary Files

File name: Supplementary Data 1
Description: Natural variation in *Rpp6907-7*. A multiple nucleotide sequence alignment of the gene with the highest sequence similarity to *Rpp6907-7* in 13 soybean varieties.

File name: Supplementary Data 2
Description: Resistance response and transgene copy number on leaves harvested from individual T1 plants challenged with *P. pachyrhizi.*

File name: Supplementary Data 3
Description: Efficacy evaluation of ASR isolates or field populations from the U.S. and Brazil.

File name: Supplementary Data 4
Description: Primers used for site-directed mutation.

File name: Supplementary Data 5
Description: Amino acid sequence of Rpp6907-4

File name: Supplementary Data 6
Description: Primers and probes used in this study.
